# Chiral nematic self-assembly of minimally surface damaged chitin nanofibrils and its load bearing functions

**DOI:** 10.1038/srep23245

**Published:** 2016-03-18

**Authors:** Dongyeop X. Oh, Yun Jeong Cha, Hoang-Linh Nguyen, Hwa Heon Je, Yong Seok Jho, Dong Soo Hwang, Dong Ki Yoon

**Affiliations:** 1 Research Center for Industrial Chemical Biotechnology, Korea Research Institute of Chemical Technology (KRICT), Ulsan 44429, Republic of Korea; 2 Graduate School of Nanoscience and Technology and KINC, KAIST, Daejeon 305-701, Republic of Korea; 3 Division of Integrative Biosciences and Biotechnology, Pohang University of Science and Technology (POSTECH), Pohang 790-784, Republic of Korea; 4 Department of Physics, Pohang University of Science and Technology; 5 Asia Pacific Center for Theoretical Physics, Pohang 790-784, Republic of Korea; 6 School of Environmental Science and Engineering, Pohang University of Science and Technology (POSTECH), Pohang 790-784, Republic of Korea

## Abstract

Chitin is one of the most abundant biomaterials in nature, with 10^10^ tons produced annually as hierarchically organized nanofibril fillers to reinforce the exoskeletons of arthropods. This green and cheap biomaterial has attracted great attention due to its potential application to reinforce biomedical materials. Despite that, its practical use is limited since the extraction of chitin nanofibrils requires surface modification involving harsh chemical treatments, leading to difficulties in reproducing their natural prototypal hierarchical structure, i.e. chiral nematic phase. Here, we develop a chemical etching-free approach using calcium ions, called “natural way”, to disintegrate the chitin nanofibrils while keeping the essential moiety for the self-assembly, ultimately resulting in the reproduction of chitin’s natural chiral structure in a polymeric matrix. This chiral chitin nanostructure exceptionally toughens the composite. Our resultant chiral nematic phase of chitin materials can contribute to the understanding and use of the reinforcing strategy in nature.

Natural hard tissues such as bones and exoskeleton are composed of organic/inorganic hybrid composites, in which the hierarchically assembled one-dimensional fillers are impregnated to provide strong mechanical properties^1^,^2^,^3^,^4^,^5^. The exoskeletons of arthropods are built from chitin nanofibrils embedded in calcium carbonate or/and protein matrices (Fig. 1a)^6^,^7^,^8^,^9^,^10^,^11^,^12^,^13^,^14^. The chitin nanofibrils (Fig. 1aII), which consist of rigid crystalline and flexible amorphous regions (Fig. 1aIII,IV), stack together to form the layered structures^15^. These structures form the basic units of the twisted plywood structure (Fig. 1aI), which is a chiral nematic phase and critical for the strong load-bearing property of the exoskeleton^5^,^11^,^12^,^13^,^14^. This property has encouraged studies on the chiral nematic shape of chitin, with the objective of reinforcing biocompatible nanocomposites. However, the harsh chemical treatment is necessary to effectively detach the individual building blocks from chitin materials hinders the reproduction of the chiral nematic phase and hence its application.

In the absence of calcium carbonates or proteins, chitin nanofibrils strongly agglomerate, not in the exploitable nanocomposite filler form, but rather in a meaningless lump due to the strong interfibrillar hydrogen bonding (H-bond) (Fig. 1b)^16^. The two most well-known chitin nanomaterials obtained using the harsh treatments are chitin nanowhiskers^9^,^17^ and chitin nanofibers (Fig. 2a,b)^18^,^19^. Both nanomaterials show no long-range ordered optical textures in polarized optical microscopy (POM) images (Fig. 2c,f). In enlarged views using scanning electron microscopy (SEM) and transmission electron microscopy (TEM), chitin nanowhiskers and nanofibers are randomly oriented (Fig. 2d,e,g,h).

Mostly, a rich amount of multivalent metals such as calcium (Ca), zinc (Zn), and iron (Fe) are found in most load-bearing chitin-based exoskeletons^6^,^8^,^12^. Although the relationship between the incorporated metals and the mechanical properties is not clearly understood, it has been reported that chitin strongly binds the metals^6^,^12^,^20^. Recently, the Tokura group reported that Ca-saturated methanol dissolves chitin at molecular level under mild conditions without strong base or acid^10^; it also shows the interaction between chitin and Ca^2+^ ion. If Ca^2+^ ions fully surround the chitin nanofibrils, the interfibrillar H-bonds could be screened.

Based on the binding between Ca^2+^ and the hydroxyl groups of chitin^20^,^21^, we developed a new strategy 1) to physically disintegrate chitin to a nanoscale 1D structure called “chitin nanowire” using our modified Ca-saturated methanol (Ca-methanol) and 2) to control 3D hierarchical structure of the chitin nanowires (Fig. 1b and 3a–c and Fig. S1). Empirically, the binding affinity of the methanol-solvated Ca^2+^ in the Ca-methanol to the chitin nanowire is comparable with the H-bond between chitin nanowires and the probability to replace the densely cross-linked H-bonds inside the chitin crystalline structure is extremely low due to the limited diffusion of methanol-solvated Ca^2+^. However, the interfibrillar region is permeable to methanol-solvated Ca^2+^, leading to the dispersion of the chitin nanowires without damage to the acetyl groups if the concentration of Ca^2+^ is sufficient to partially replace the interfibrillar H-bond. After the disassembly process, Ca^2+^ ions are removed through solvent exchange using three different solvents: isopropanol (IPA), methanol, and deionized (DI) water: DI water shows the highest binding affinity to Ca^2+^, while IPA shows the lowest^22^. Thus, reassembled chitin nanowires generate different phases in IPA gel, methanol gel, and hydrogel. In IPA and methanol gel states, a nematic liquid crystal (LC) (**N**) phase is generally observed (Fig. 3d–f), while in hydrogel state, it shows a chiral nematic LC (N*) phase (Fig. 3g–i, for an illustration see the hydrogel panel in Fig. 1b).

## Results and Discussion

**Figure 1:**
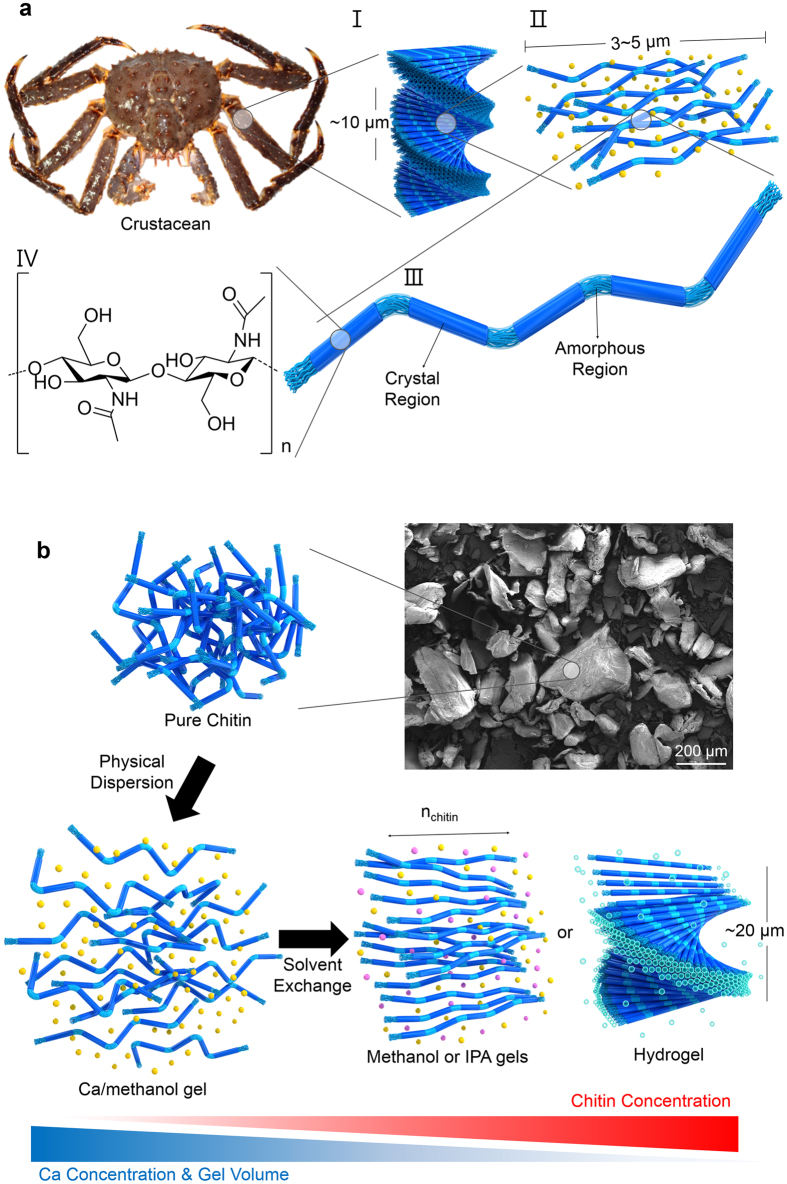
Hierarchically-ordered chitin microstructure in an arthropod cuticle. (**a**) (I) Plywood structure of chitin nanofibrils, (II) chitin nanofibrils in the matrix (CaCO_3_ or proteins), (III) crystalline and amorphous domains of chitin nanofibril structure, and (IV) chitin structural formula. (**b**) Calcium-saturated methanol disintegrates chitin nanofibrils with minimal chemical modification, generating a Ca-methanol gel (disordered) (*bottom-left* panel). Ca^2+^ are removed from the Ca-methanol gel by washing with alcohol (methanol or IPA) and DI water, thus generating alcohol gels (methanol gel or IPA gel) in the **N** phase (*bottom-middle* panel) and a hydrogel in the N* phase (*bottom-right* panel). The yellow, pink, and blue beads represent three different types of solvent molecules: methanol-solvated Ca^2+^, alcohol (methanol or IPA), and water.

The nanostructures of chitin nanowires at each gel state were visualized by POM and cryogenic TEM (Cryo-TEM) (Fig. 3). Considering the long axis of chitin nanowires as director vector, **n**_**chitin**_ (Fig. 1b), POM can show the orientation of chitin nanowires, i.e. the image is dark when molecules are disordered or **n**_**chitin**_ is parallel to either of polarizer or analyser. POM with the full-wavelength (530 nm) retardation plate can show the detailed orientation, with the image showing a magenta colour when the sample is isotropic or disordered and blue/yellow domains when **n**_**chitin**_ is parallel/perpendicular, respectively, to the slow axis of the retardation plate. In the Ca-methanol gel, there are no specific morphologies in POM images (Fig. 3a,b) due to the randomly oriented or disordered chitin nanowires shown in the TEM image (Fig. 3c). POM images of the IPA gel show a well-oriented domain in a large area (~mm^2^), in which the **N** phase is found^9^. This is confirmed by TEM (Fig. 3f), which shows one-dimensionally aligned chitin nanowires. The chitin nanowire-hydrogel forms a N* phase, showing the fingerprint patterns in POM images, in which alternating bright and dark lines are present (Fig. 3g). The periodicity of the stripe pattern is given by the helical pitch P/2 (Fig. 1b), showing a pitch P of ~10 to 40 μm with a broad distribution due to the irregular out-of plane arrangement of chitin nanowires. A corresponding TEM image shows a Bouligand-type organization of chitin nanowires (Fig. 3i), which is typically found in the N* phase^23^,^24^,^25^,^26^. The counter-clockwise direction of the morphologies means the chitin nanowires assemble into left-handed helical structures in the gel state^12^,^26^,^27^.

**Figure 2:**
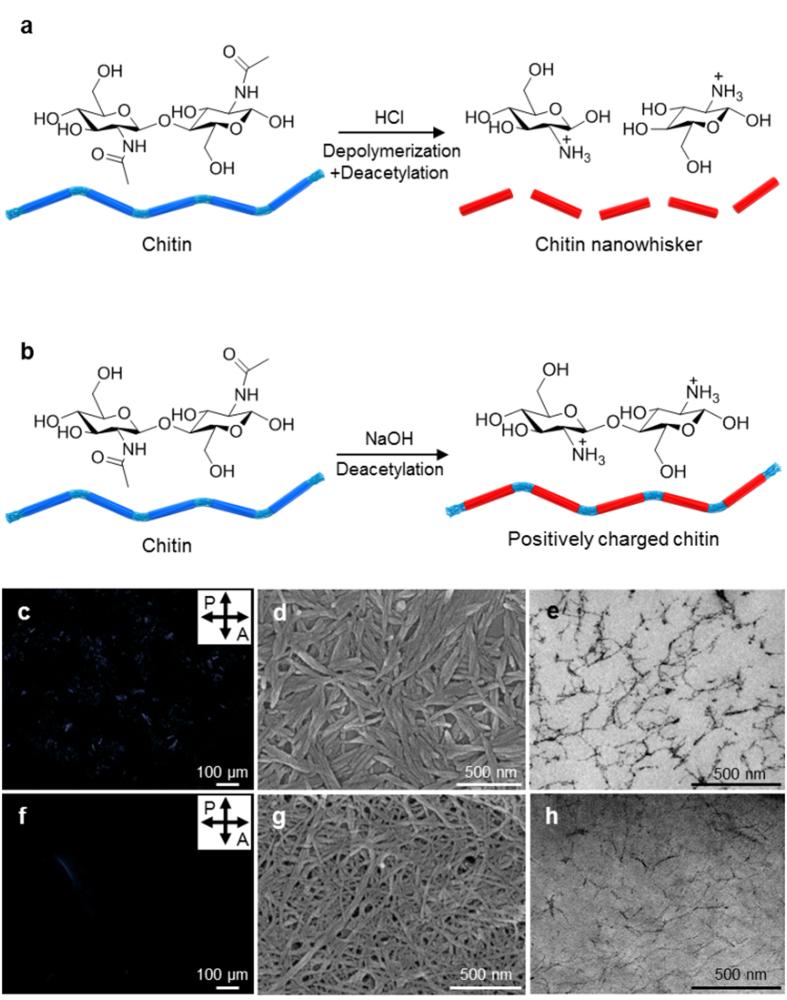
Chemically modified chitin nanomaterials. (**a**) nanowhisker and (**b**) nanofiber. POM images of (**c**) nanowhisker and (**f**) nanofiber solutions (aq). SEM images of (**d**) dried nanowhisker and (**g**) dried nanofiber. Cryo-TEM images of (**e**) nanowhisker and (**h**) nanofiber solutions (aq).

**Figure 3:**
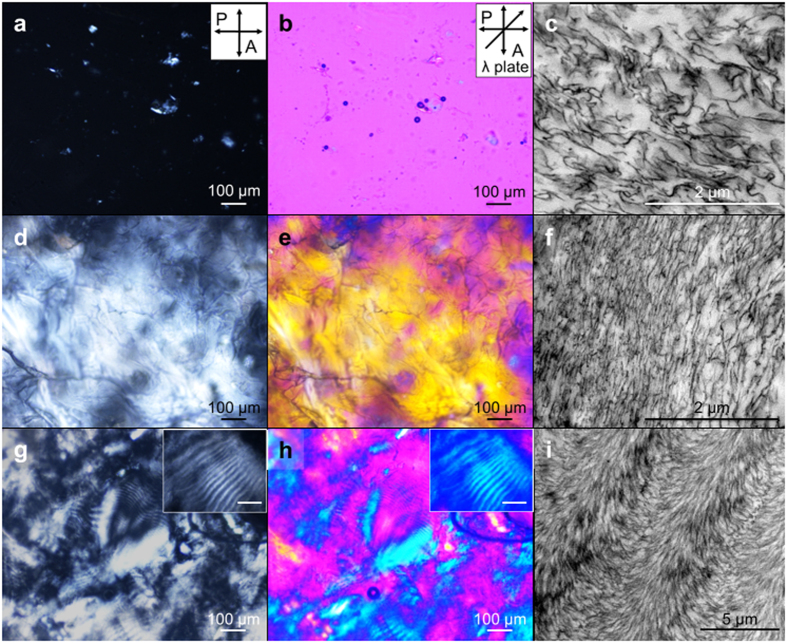
Morphological change of chitin nanowires by solvent exchange. POM (left and centre panels) and TEM (right panels) images of Ca-methanol gel (**a**–**c**), IPA gel (**d**–**f**), and hydrogel (**g**–**i**, with an inset scale of 50 μm).

These **N** and N* phases are different from the natural states in terms of the domain size^11^,^12^. Unlike the previously reported N* phase of highly concentrated nanocellulose in silica^23^, our resultant chiral hydrogel shows a finite domain size of 200–600 μm (Fig. 3g,h), which may be caused by an energetic penalty of having a large domain size. Here, the charge level of the composite can be divided into 3 classes; i) neutral, when the natural chitin materials are partially cationic are mixed with negatively charged matrix materials including ions and proteins;^27^ ii) slightly positively charged chitin materials^28^ made via natural way with minimal surface deacetylation (Fig. 1b), and iii) strongly positively charged chitin materials made by severe surface deacetylation (Fig. 2). In our resultant N* phase, due to the long-range nature of electrostatic interactions, the energetic penalty can be increased as the domain grows much faster than the energetic gain of having H-bond, which is a short-ranged interaction and thus grows linearly. At a certain size of the domain, both energy loss and gain compensate each other and the growing stops, resulting in finite N* domains. Contrary to this, chitin materials in nature can nucleate and grow to form large or infinite-sized domains without any energetic penalty, despite the charged nature of chitin, since in nature compensating opposite charges are present nearby^27^, and charge neutrality is valid locally.

Since chitin has poor solubility in water and alcohol, the solvent exchange in absence of Ca^2+^ results in agglomerated structures. The binding affinity of Ca^2+^ is the highest to water molecule and the lowest to IPA, thus, Ca^2+^ binds less to chitin in water than in IPA. The chitin concentration is the highest for hydrogel and the lowest for IPA gel in an inverse order of the Ca^2+^ concentration in the equilibrium condition (Fig. S2). Therefore, the amorphous regions of the chitin nanowires are more hydrated and swollen than the rigid crystalline parts in the chitin hydrogel (Fig. S3), not leading to the parallel stacked chitin nanowires but rather to its twisted form (Fig. 3g–i). In this way, the helical pitch of the chitin nanowires in the N* phase can be controlled through the water content of the hydrogel (Fig. S4). Evaporation of water generates water flow, which puts hydrodynamic pressure on the nanowires to form more compactly stacked structures. This also reduces the thickness of the hydration shells of amorphous and crystalline domains. Thus, the helical pitch in the N* phase can be decreased. Meanwhile, the thickness of IPA solvation shells in both crystalline and amorphous regions is not substantially different, leading to an **N** phase.

**Figure 4:**
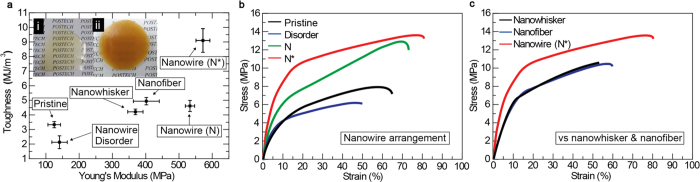
Mechanical reinforcing effects of chiral nematic chitin nanowires to epoxy resin. (**a**) Tensile Young’s modulus and toughness of pristine epoxy and chitin epoxy composites. The inset pictures (i) and (ii) show pristine epoxy and chiral nematic chitin epoxy composite films, respectively. (**b**) Tensile stress-strain curves of epoxy composites with disordered, nematic (N), and chiral nematic (N*) phases of chitin nanowires. (**c**) Tensile stress-strain curves of chitin nanowhisker and nanofiber epoxy composites are compared.

To demonstrate the versatility of our method, the gel-based composite patches were manipulated. The process is simple, and three gel states of chitin nanowires including Ca-methanol gel (disordered), IPA gel (**N**), hydrogel (N*) were embedded in an epoxy resin, similar to sample preparation for Cryo-TEM (Fig. 3c,f,i)^29^. Then, we studied the mechanical properties of each type of gel structure. At a fixed chitin content of ~50 g/L in the patches, the Young’s modulus (*E*; stiffness) and the toughness (amount of energy stored per unit volume (J/m^3^) during stretching until failure) were measured for each gel (Fig. 4). Both *E* and toughness increase as the higher ordered structures are formed (Fig. 4a). However, even though we could not find any substantial difference in *E* between the **N** and N* phases, the toughness of N* is much larger than that of **N** (Fig. 4a,b). In the macroscopic perspective, the finite **N** and N* domains are randomly orientated in the patches, meaning that *E* does not depend much on the hierarchical structures of the chitin nanowires embedded in the patches. During deformation, the N* domains redirects the crack direction and prevents crack propagation in the composite^11^,^12^,^14^,^30^, giving rise to the notable enhancement in the toughness. It is practically difficult to remove high concentrations of CaCl_2_ (~670 g/L) from the Ca-methanol using acetone at −80 °C. Therefore, the epoxy resin was not effectively impregnated in mineral-containing chitin nanowires, and the poor interfacial adhesion between the CaCl_2_ aggregate and the epoxy resin may explain why the composite from the Ca-methanol gel has the weakest mechanical properties.

To confirm the morphological characteristics and the chemical intactness of chitin nanowires, the chitin nanowhiskers and nanofibers were also impregnated in the epoxy resin with the same chitin content of ~50 g/L. Both nanowhisker and nanofiber composites gave much poorer tensile properties in terms of *E* and toughness when compared to our resultant nanowire patches (Fig. 4c). This shows that the degradation of chitin molecules during the chemical modification softens chitin nanowhiskers and nanofibers, and the resulting high density charges prevent the formation of high ordered structures.

Our chiral nematic chitin nanowires can be applied to the biocompatible composite of polyethylene glycol (PEG) hydrogel (Figs S5 and S6). The tensile properties of the PEG hydrogel patches also show that the chitin nanowire based patch has the highest toughness.

## Conclusions

In summary, we tried to understand and reproduce the chiral plywood structures using chitin nanowires extracted from the crustacean shell via natural way. A chiral nematic arrangement of minimally modified chitin, an unprecedented hierarchical assembly of nanofibril natural polymers in aqueous phase, was obtained. The mechanical properties of the resultant composites demonstrated changes according to the conformation of chiral nanomaterials. Our resultant chiral chitin nanowires can contribute to the understanding of the load bearing systems in nature and potentially enable new biocompatible reinforcing applications.

## Additional Information

**How to cite this article**: Oh, D. X. *et al*. Chiral nematic self-assembly of minimally surface damaged chitin nanofibrils and its load bearing functions. *Sci. Rep.* **6**, 23245; doi: 10.1038/srep23245 (2016).

## Supplementary Material

Supplementary Information

## References

[b1] Tang Z., Kotov N. A., Magonov S. & Ozturk B. Nanostructured Artificial Nacre. Nat. Mater. 2, 413–418 (2003). 12764359 10.1038/nmat906

[b2] Deville S., Saiz E., Nalla R. K. & Tomsia A. P. Freezing as a Path to Build Complex Composites. Science 311, 515–518 (2006). 16439659 10.1126/science.1120937

[b3] Erb R. M., Libanori R., Rothfuchs N. & Studart A. R. Composites Reinforced in Three Dimensions by Using Low Magnetic Fields. Science 335, 199–204 (2012). 22246772 10.1126/science.1210822

[b4] Capadona J. R., Shanmuganathan K., Tyler D. J., Rowan S. J. & Weder C. Stimuli-Responsive Polymer Nanocomposites Inspired by the Sea Cucumber Dermis. Science 319, 1370–1374 (2008). 18323449 10.1126/science.1153307

[b5] Zimmermann E. A. . Mechanical adaptability of the Bouligand-Type Structure in Natural Dermal Armour. Nat. comm. 4, 1–7 (2013). 10.1038/ncomms3634 24129554

[b6] Vincent J. F. V. Arthropod Cuticle: A Natural Composite Shell System. Composites Part A 33, 1311–1315 (2002).

[b7] Miserez A., Schneberk T., Sun C., Zok F. W. & Waite J. H. The Transition from Stiff to Compliant Materials in Squid Beaks. Science 319, 1816–1819 (2008). 18369144 10.1126/science.1154117 PMC2754134

[b8] Gordon L. M. & Joester D. Nanoscale chemical tomography of buried organic-inorganic interfaces in the chiton tooth. Nature 469, 194–197 (2011). 21228873 10.1038/nature09686

[b9] Nguyen T. D., Shopsowitz K. E. & MacLachlan M. J. Mesoporous Silica and Organosilica Films Templated by Nanocrystalline Chitin. Chem. Euro. J. 19, 15148–15154 (2013). 10.1002/chem.201301929 24150881

[b10] Tamura H., Nagahama H. & Tokura S. Preparation of Chitin Hydrogel under Mild Conditions. Cellulose 13, 357–364 (2006).

[b11] Weaver J. C. . The Stomatopod Dactyl Club: A Formidable Damage-Tolerant Biological Hammer. Science 336, 1275–1280 (2012). 22679090 10.1126/science.1218764

[b12] Nikolov S. . Revealing the Design Principles of High‐Performance Biological Composites Using Ab Initio and Multiscale Simulations: The Example of Lobster Cuticle. Adv. Mater. 22, 519–526 (2010). 20217746 10.1002/adma.200902019

[b13] Bar-On B., Barth F. G., Fratzl P. & Politi Y. Multiscale Structural Gradients Enhance the Biomechanical Functionality of the Spider Fang. Nat. comm. 5, 1–8 (2014). 10.1038/ncomms4894 PMC4050259 24866935

[b14] Grunenfelder L. K. . Bio-Inspired Impact-Resistant Composites. Acta Biomater. 10, 3997–4008 (2014). 24681369 10.1016/j.actbio.2014.03.022

[b15] Nishino T., Matsui R. & Nakamae K. Elastic Modulus of the Crystalline Regions of Chitin and Chitosan. J. Polym. Sci. Part B Polym. Phys. 37, 1191–1196 (1999).

[b16] Ifuku S. . Fibrillation of Dried Chitin into 10–20nm Nanofibers by a Simple Grinding Method under Acidic Conditions. Carbohydr. Polym. 81, 134–139 (2010).

[b17] Zeng J. B., He Y. S., Li S. L. & Wang Y. Z. Chitin whiskers: An overview. Biomacromolecules 13, 1–11 (2011). 22148591 10.1021/bm201564a

[b18] Das P. . Tough and Catalytically Active Hybrid Biofibers Wet-Spun from Nanochitin Hydrogels. Biomacromolecules 13, 4205–4212 (2012). 23102411 10.1021/bm3014796

[b19] Kato Y., Kaminaga J., Matsuo R. & Isogai A. TEMPO-Mediated Oxidation of Chitin, Regenerated Chitin and N-Acetylated Chitosan. Carbohydr. Polym. 58, 421–426 (2004).

[b20] Gyliene O., Rekertas R. & Šalkauskas M. Removal of free and complexed heavy-metal ions by sorbents produced from fly (Musca domestica) larva shells. Water Res. 36, 4128–4136 (2002). 12405421 10.1016/s0043-1354(02)00105-7

[b21] Cook W. J. & Bugg C. E. Calcium Binding to Galactose. Crystal Structure of a Hydrated α-Galactose-Calcium Bromide Complex. J. Am. Chem. Soc. 95, 6442–6446 (1973). 4733397 10.1021/ja00800a048

[b22] Taniewska-Osinska S. & Barczynska J. Enthalpy of Solution of Calcium Chloride in Aqueous Mixtures of Methanol, Ethanol and Propan-1-ol at 298.15 K. J. Chem. Soc. Faraday Trans. 80, 1409–1414 (1984).

[b23] Shopsowitz K. E., Qi H., Hamad W. Y. & MacLachlan M. J. Free-Standing Mesoporous Silica Films with Tunable Chiral Nematic Structures. Nature 468, 422–425 (2010). 21085176 10.1038/nature09540

[b24] Park J. H. . Macroscopic Control of Helix Orientation in Films Dried from Cholesteric Liquid‐Crystalline Cellulose Nanocrystal Suspensions. ChemPhysChem 15, 1477–1484 (2014). 24677344 10.1002/cphc.201400062

[b25] Liu Q., Campbell M. G., Evans J. S. & Smalyukh I. I. Orientationally Ordered Colloidal Co‐Dispersions of Gold Nanorods and Cellulose Nanocrystals. Adv. Mater. 26, 7178–7184 (2014). 25164198 10.1002/adma.201402699

[b26] Nguyen T. D. & MacLachlan M. J. Biomimetic Chiral Nematic Mesoporous Materials from Crab Cuticles. Adv. Opt. Mater. 2, 1031–1037 (2014).

[b27] Fernandez J. G. & Ingber D. E. Bioinspired Chitinous Material Solutions for Environmental Sustainability and Medicine. Adv. Funct. Mat. 23, 4454–4466 (2013).

[b28] Majtán J. . Isolation and Characterization of Chitin from Bumblebee (Bombus Terrestris). Int. J. Biol. Macromol. 40, 237–241 (2007). 16949663 10.1016/j.ijbiomac.2006.07.010

[b29] Hoiczyk E. & Baumeister W. Envelope Structure of Four Gliding Filamentous Cyanobacteria. J. Bacterial. 177, 2387–2395 (1995). 10.1128/jb.177.9.2387-2395.1995 PMC176896 7730269

[b30] Andersons J. & König M. Dependence of Fracture Toughness of Composite laminates on Interface Ply Orientations and Delamination Growth Direction. Comp. Sci. Technol. 64, 2139–2152 (2004).

