# Supplementary material for: Chiral nematic self-assembly of minimally surface damaged chitin nanofibrils and its load bearing functions
Source: Sci Rep. 2016 Mar 18;6:23245. doi: 10.1038/srep23245 (PMC4796797; doi:10.1038/srep23245)
Supplement: Supplementary Information [file srep23245-s1.pdf]

Supplementary Information

Chiral nematic self-assembly of minimally surface damaged chitin nanofibrils and its load bearing functions

Dongyeop X. Oh[1,+], *Yun Jeong Cha*[2,+], *Hoang-Linh Nguyen*[3], *Hwa Heon Je*[3], *Yong Seok Jho*[4,5], *Dong Soo Hwang*[3,6,*], *& Dong Ki Yoon*[2,*]

[1]Research Center for Industrial Chemical Biotechnology, Korea Research Institute of Chemical Technology (KRICT), Ulsan 44429, Republic of Korea

[2]Graduate School of Nanoscience and Technology and KINC, KAIST, Daejeon 305-701, Republic of Korea

[3]Division of Integrative Biosciences and Biotechnology, Pohang University of Science and Technology (POSTECH), Pohang 790-784, Republic of Korea

[4]Department of Physics, Pohang University of Science and Technology, [5]Asia Pacific Center for Theoretical Physics, Pohang 790-784, Republic of Korea

[6]School of Environmental Science and Engineering, Pohang University of Science and Technology (POSTECH), Pohang 790-784, Republic of Korea

[+]These authors contributed equally to this work.

*Correspondence should be addressed to Dong Soo Hwang (e-mail: dshwang@postech.ac.kr) & Dong Ki Yoon. (e-mail: nandk@kaist.ac.kr)

Methods

Materials

Calcium chloride di-hydrate ($CaCl_2.H_2O$) and methanol were purchased from Junsei Chemical (Japan). Chitin powder, epoxy embedding medium kit, and polyethylene glycol diacrylate (PEGDA; 700 g/mol) were purchased from Sigma-Aldrich. IPA and 2-hydroxy-4'-(2-hydroxyethoxy)-2 methylpropiophenone (photo-initiator; PI) were purchased from TCI Chemical (Japan). All aqueous solutions were prepared using deionized (DI) water.

Physical disintegration of chitin to chitin nanowire: Ca-methanol gel

Ca-methanol was prepared as follows[10]. Calcium chloride di-hydrate (428 g) and DI water (30 ml) were added to methanol (470 ml). The mixture was refluxed at 150 °C for 6 h. To produce Ca-methanol gel, chitin powder (8 g) was added to the Ca-methanol (400 ml), then the mixture was refluxed in at 150 °C for 6 h and cooled down at room temperature.

Solvent exchange of Ca-methanol gel to produce hydrogel, methanol gel, and IPA gel

Ca^{2+} ions were removed from the Ca-methanol gel via solvent-exchange with three different solvents: DI water, methanol, and IPA[10]. The Ca-methanol gel was immersed in excess volume of each exchange solvent with vigorous stirring for 12 h, then filtrated by nylon cloth to collect the chitin gel precipitate. The chitin gel precipitates were centrifuged at 8000 rpm for 15 min to remove excess of solvent. Consequently, hydrogel (=chiral chitin nanowire hydrogel), methanol gel, and IPA gel were obtained.

Chitin and calcium concentration

Chitin concentration (weight per volume; g/L) of each gel was evaluated as follows. Ten millilitre of each gel was incubated in EDTA buffer at 37 °C for 48 h with shaking and vigorously washed with DI water. The insoluble part was dried at 100 °C in a vacuum oven for 24 h. And then, the dry weight of the insoluble part was measured.

Calcium concentration (weight per volume; g/L) of each gel was evaluated as follows. Ten millilitre of each gel was dried at 100 °C in a vacuum oven for 24 h. And then, the weight of the dried sample was measured. Calcium weight in the dry sample was investigated using an inductively coupled plasma atomic emission spectroscopy (ICP-AES) on an IRIS Advantage 1000.

Preparation of chitin nanowhisker and nanofiber

Chitin nanowhisker was prepared as follows.[17] Chitin powder (20 g) was added into 3 N HCl (400 ml). And then, the mixture was refluxed at 150 °C for 6 h. The solution was purified 3 times with DI water using a series of dilution-centrifugation-decantation process. The suspension was dialyzed overnight in DI water, then lyophilized. The lyophilized chitin nanowhisker was re-dispersed in 50 mM acetate buffer (pH 4) via ultra-sonication.

Chitin nanofiber was prepared as follows[16-18]. Chitin powder (20 g) was suspended in 20 wt% NaOH (*aq*) (400 ml), then refluxed at 150 °C for 6 h. The wet sample was vigorously washed with DI water, then dispersed in DI water to be ~1.3 wt %. Drops of acetic acid was added into the chitin suspension to adjust the pH value to ~4. For disintegration, the chitin suspension passed through a high performance grinder (MKCA6−3; Masuko Sangyo Co., Ltd.) with the rotation speed of 1500 rpm and a clearance gauge of −1.5. The chitin nanofiber suspension was dialyzed overnight in DI water, then lyophilized. The lyophilized chitin nanofiber was re-dispersed in 50 mM acetate buffer (pH 4) by ultra-sonication.

Cryo-TEM

Each sample was placed on gold-plated membrane carriers[29, 31]. The sample-containing carrier was loaded into a Leica EM HPM100 (Germany) and rapidly frozen with liquid nitrogen under high pressure of 2100 bar. The sample was freeze-substituted with 0.2% uranyl acetate-containing dry acetone at −80 °C for 3 days. The sample was warmed from −80 °C to −20 °C, immersed in pure acetone for 3 day, infiltrated with acetone/epoxy resin precursor (epoxy embedding medium kit) mixtures (4:1 for 75 min and 2:1 for 2 h) at −20 °C, and infiltrated with the pure epoxy resin precursor for 12 h at room temperature. The sample was cured at 70 °C for 24 h. The resin was cut into 200 nm-sections using Leica EM UC7 (Germany) and applied onto copper grids. All TEM images were recorded using a JEOL JEM 1011 transmission electron microscope. The resin precursor contains epoxy embedding medium (20 ml), 2-dodecenylsuccinic anhydride (9 ml), methyl nadic anhydride (12 ml), and 2,4,6-tris(dimethylaminomethyl)phenol (1 ml).

Equilibrium water content (EWC) and equilibrium alcohol content (EAC)

To characterize the degree of water and alcohol absorption, the EWC and EAC were measured[32]. Dry chitin (or regenerated chitin) was weighed to an accuracy of 10^{-4} g and immersed in water (or isopropanol) for 1 day. And then, the immersed film was weighed

again after removal of excess liquid. EWC or EAC were calculated using the following equation:

$$\frac{Wt - W0}{Wt} X\ 100$$

W_0 is the weight of the sample before immersion and W_t is the weight of the sample after immersion at time t when the weight of the sample reaches the equilibrium state.

The regenerated chitin was prepared as follows. One gram of chitin powder was dissolved in nine gram of 1-ethyl-3-methylimidazolium acetate ([C_2mim] [OAc], an ionic liquid) at 100 ℃ for 6 h with stirring. The solution was cooled down in ambient conditions for 1 day to form a gel. The gel was soaked in excess of water for 6 h and, then washed with distilled water.

Chitin nanowire-embedded epoxy composites

Ca-methanol gel, IPA gel, and hydrogel were rapidly frozen with liquid nitrogen[29, 31]. Each sample was freeze-substituted with pure acetone at −80 ℃ for 3 days and at −20 ℃ for 3 days. The sample was infiltrated with acetone-epoxy resin precursor mixtures (4:1 for 1 day and 2:1 for 1 day) at −20 °C, and infiltrated with the pure epoxy resin precursor at room temperature for 12 h. The chitin/epoxy resin precursor suspension was centrifuged at 8000 rpm for 20 min. The desire amount of the epoxy resin precursor was added into the precipitate-containing bottle to adjust chitin contration. And then, the mixture was homogenized with a domestic blender and an ultra-sonicator. The suspension was cured at 70 ℃ for 24 h. The epoxy resin precursor contains epoxy embedding medium (20 ml), 2-dodecenylsuccinic anhydride (22 ml), methylnadic anhydride (2 ml), and 2,4,6-tris(dimethylaminomethyl)phenol (0.6 ml).

Nanofiber- and nanowhisker-embedded epoxy composites

Nanofiber (or nanowhisker)-dispersed solution at the chitin concentration of ~20 g/L was rapidly frozen with liquid nitrogen. The sample was freeze-substituted with pure acetone at −80 ℃ for 3 days and at −20 ℃ for 3 days. The sample was infiltrated with acetone-epoxy resin precursor mixtures (4:1 for 1 day and 2:1 for 1 day) at −20 ℃, and infiltrated with the pure epoxy resin precursor at room temperature for 12 h. The chitin/epoxy resin precursor suspension was centrifuged at 800 rpm for 20 min. The desire amount of the epoxy resin precursor was added into the precipitate-containing bottle to adjust chitin concentration. And then, the mixture was homogenized with a domestic blender and an ultra-sonicator. The suspension was cured at 70 ℃ for 24 h. The epoxy resin precursor contains epoxy embedding

medium (20 ml), 2-dodecenylsuccinic anhydride (22 ml), methylnadic anhydride (2 ml), and 2,4,6-tris(dimethylaminomethyl)phenol (0.6 ml).

Chiral chitin nanowire-embedded PEG hydrogel composites

Chiral chitin nanowire-embedded polyethylene glycol (PEG) hydrogels were prepared[33]. PEGDA is a precursor of PEG hydrogel, and PI is an UV-responsive cross-liking agent of the PEGDA. The chiral chitin nanowire hydrogel has chitin concentration of ~60 g/L. Desired amount of water, PEGDA, and PI were added into the chiral chitin nanowire hydrogel: PEGDA (150 g/L), PI (1.5 g/L), and chitin concentration (0, 5, 10, or 20 g/L). And then, the mixtures were vigorously homogenized using a domestic blender and an ultra-sonicator. The homogenized suspensions were incubated at 4 ℃ for 3 days for PEGDA infiltration, then cured by 15 min UV irradiation.

Nanowhisker- and nanofiber- embedded PEG hydrogel composites

Nanowhisker and nanofiber-embedded PEG hydrogels were prepared as follows[33]. Desired amount of PEGDA, PI, and lyophilized chitin nanomaterial were added into 50 mM acetic acid buffer: PEGDA (150 g/L), PI (1.5 g/L), and chitin concentration (20 g/L). The mixtures were vigorously homogenized using a domestic blender and an ultra-sonicator. The homogenized suspensions were incubated at 4 ℃ for 3 days for PEGDA infiltration, then cured by 15 min UV irradiation.

XRD

The crystal structure of chitin and chiral chitin nanowire was studied using wide-angle X-ray diffraction experiment. The experiment was conducted on an X-ray diffractometer (XRD) (D/MAX-2500/PC, Rigaku, Japan), in which a 40 kV/100 mA Ni-filtered Cu Kα radiation was employed. The WRD patterns were recorded in the region of a scattering angle of 5° to 40° with a scanning speed of 4°/min.

Tensile tests

1) Chitin/epoxy composites

The tensile properties of the chitin nanowire/epoxy composites were measured on a universal tensile tester (UTS, Instron, Norwood, UK). All samples were cut into the following dog-bone shape.

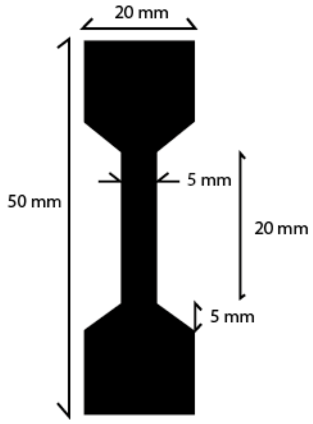

Each sample was clamped onto the grips and loaded with a constant strain rate of 5 mm/min till failure. To calculate the tensile stress (σ, MPa, load per unit cross-sectional area) on the films, thickness and width of the films were determined using a micrometer before the test. In the strain-stress curve, Young's modulus was determined as the initial slope of the curve at 0.2% strain and toughness was determined as the area under the curve until its failure.

2) Chitin PEG hydrogel composites

The tensile properties of the chitin/PEG hydrogel composites were measured on a universal tensile tester (UTS, Instron, Norwood, UK). All PEG hydrogels were cut into 30 mm × 5 mm × 4 mm cuboid shape. Each sample was clamped onto the grips with 10 mm distance and loaded with a constant strain rate of 5 mm/min till failure. To calculate the tensile stress (σ, MPa, load per unit cross-sectional area) on the films, thickness and width of the films were determined using a micrometer before the test. In the strain-stress curve, Young's modulus was determined as the initial slope of the curve at 0.2% strain and toughness was determined as the area under the curve until its failure.

Supplementary text

EWC and EAC of pure chitin and regenerated chitin

Chitin is neither meltable nor soluble in most solvents. Only solvents, such as NaOH/urea (*aq*) and ionic liquids, have been found to successfully dissolve chitin at molecular level[32]. Re-solidified chitin from those of chitin solutions is called "regenerated chitin", which have low crystallinity, in other word high content of amorphous region. Thus, the EWC data (Fig.S4) suggests that the crystalline region of chitin is less hydrated than the amorphous domain of chitin. However, the difference between EAC values of pure chitin and regenerated chitin was much lower than the difference between EWC values.

Tensile properties of the chitin nanowire-embedded PEG hydrogel.

The chiral chitin nanowire was impregnated into the PEG hydrogel (Fig. S5). To investigate how the morphology of the chitin nanomaterials affects their reinforcing property, nanowhisker and chitin nanofiber were also impregnated into the PEGDA[33]. At the same chitin concentration (20 g/L), there's no remarkable difference in E values for all of three types of chitin nanomaterials-embedded PEG hydrogels, while the toughness is much varied with the chiral arrangement of the building blocks (Fig. S6). Thus, the toughness relies on the conformation of the chitin building blocks, related with the enduring energy against the rupturing. In details, the chiral nematic structure of chitin nanowires has the multi-hierarchal arrangement of the chiral building blocks, which prevent the formation of micro-cracks and macro-crack propagation[5, 12, 14, 30]. And reasonably the stiffness and toughness of the hydrogels increased with the chiral chitin nanowire concentration.

Supplementary figures

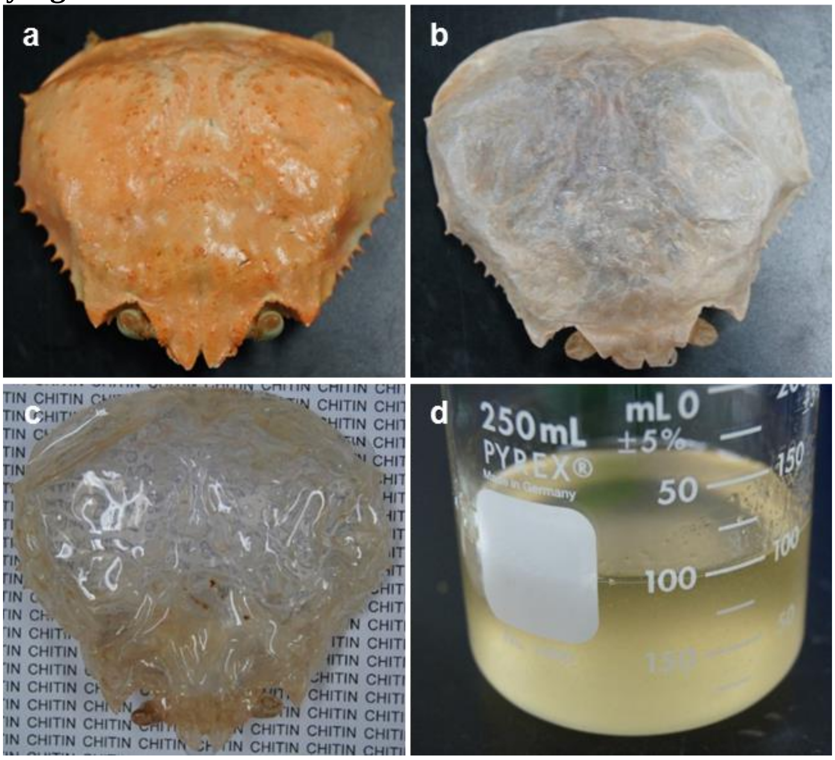

Figure S1. (a) A crab shell and (b) purified chitin skeleton from the crab shell. The chitin skeleton was immersed in Ca-methanol at (c) 70 ℃ and (d) 150 ℃.

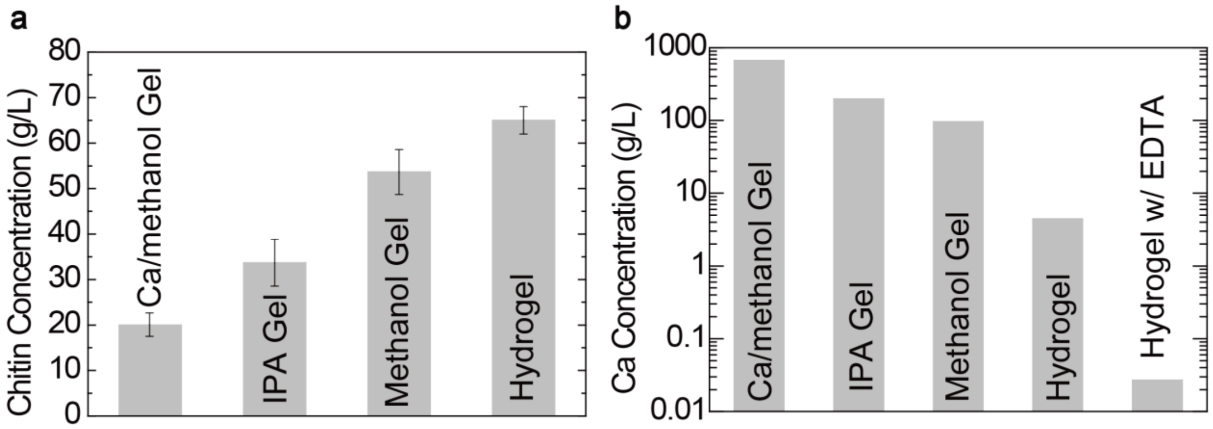

Figure S2. (a) Chitin concentration (weight per volume, g/L) of Ca/methanol gel, IPA gel, methanol gel, and hydrogel. Each value and error bar represents the mean of quadruplicate samples and its standard deviation. (b) Calcium concentration (weight per volume, g/L) of Ca-methanol, IPA gel, methanol gel, hydrogel, and EDTA-treated hydrogel from the inductively coupled plasma spectroscopy (ICP).

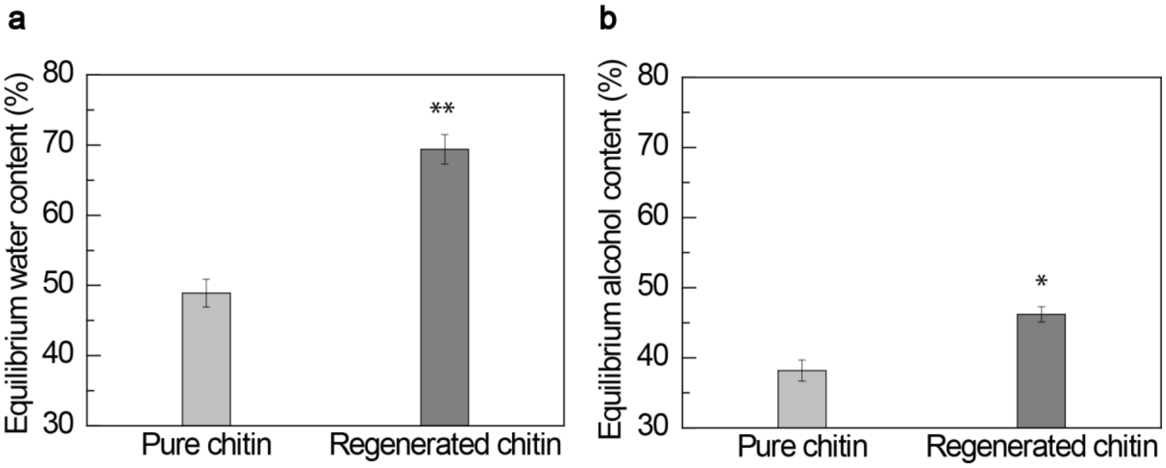

Figure S3. Equilibrium water content (EWC) and equilibrium alcohol content (EAC) of pure chitin and regenerated chitin after immersion in solvents, (a) water and (b) isopropanol (IPA) respectively, for 1 day. [n=3; mean ± the standard error of the mean; *, $P<0.05$; **, $P<0.005$)]

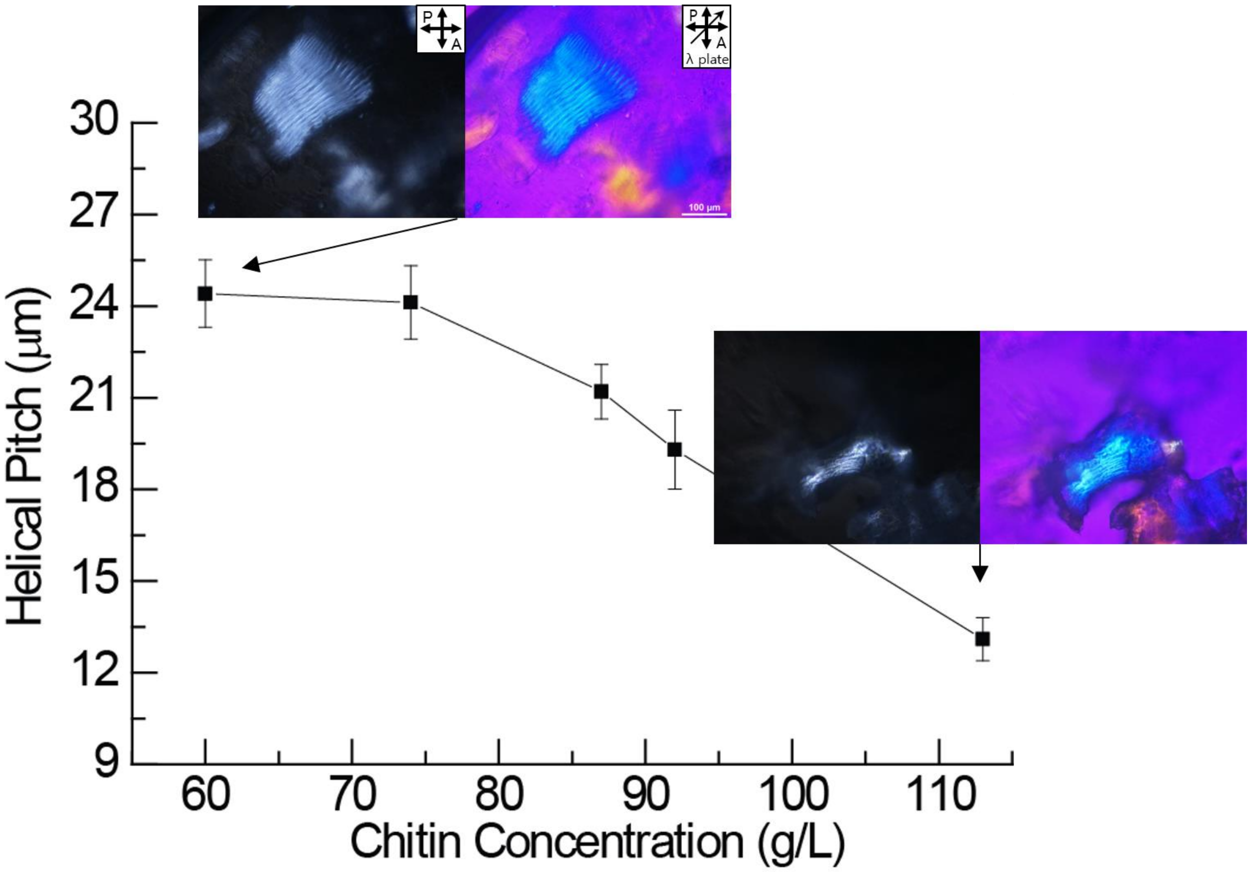

Figure S4. Helical pitch of chiral nematic chitin nanowire hydrogel depending on chitin concentration during water evaporation. Each value and error bar represents the mean of triplicate samples and its standard deviation.

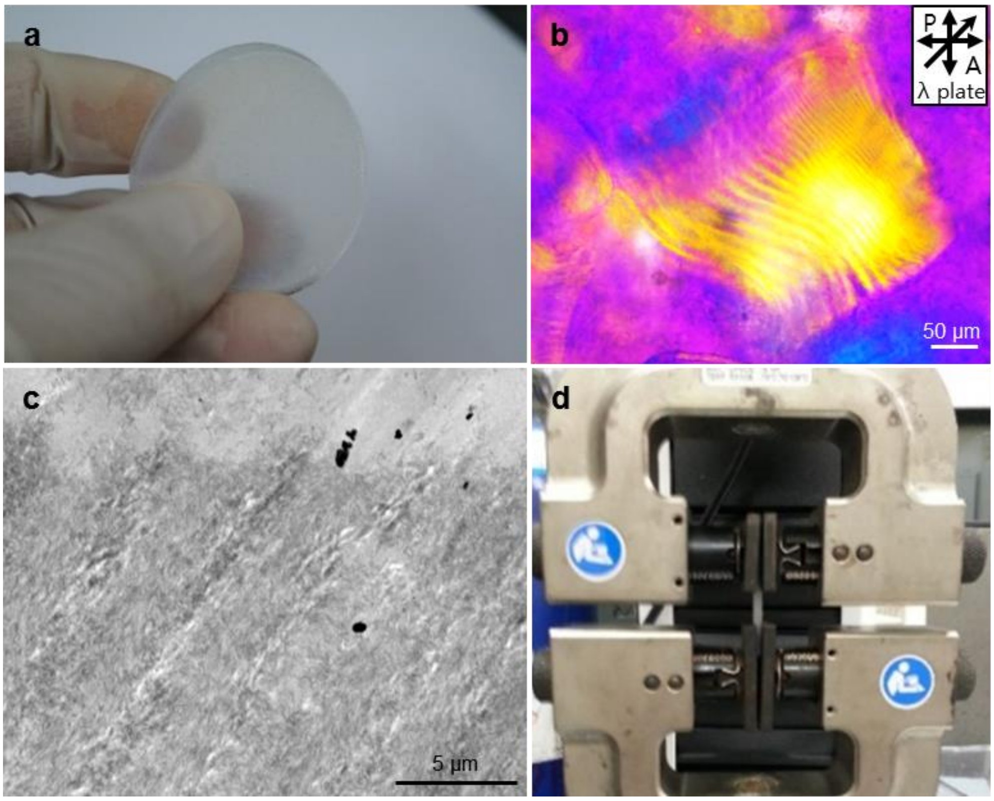

Figure S5. (a) Optical camera, (b) polarized optical microscopy, and (c) Cryo-TEM images of chiral nematic nanowire PEG hydrogel patch. (d) Experimental set-up for the tensile tests.

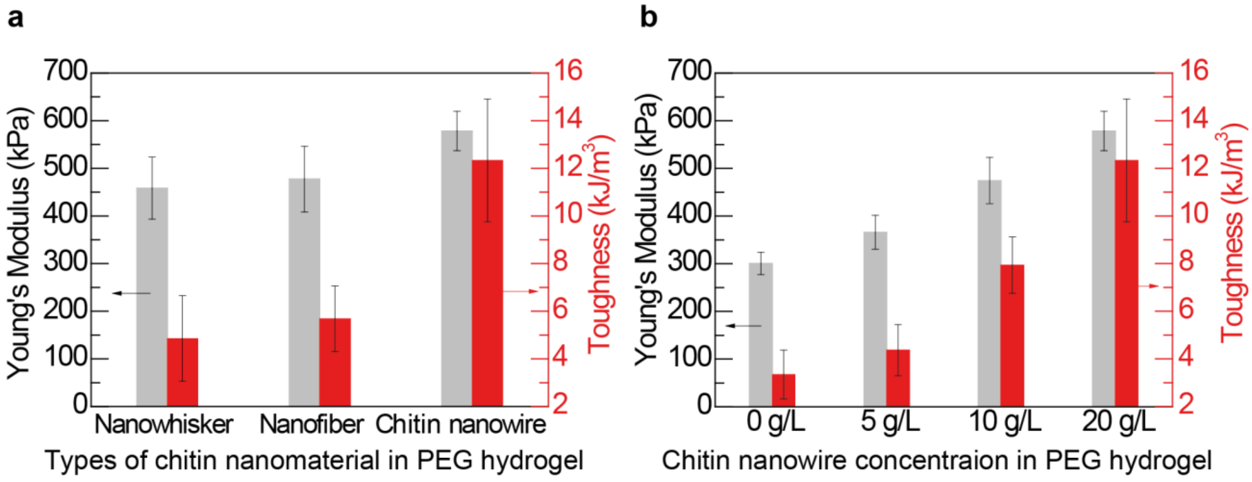

Figure S6. Tensile Young's modulus (*E*) and toughness of chitin nanomaterial-embedded PEG hydrogel patches depends on (a) types of chitin nanomaterials at the same chitin concentration (~20 g/L) and (b) chiral chitin nanowire concentration in the PEG hydrogel. Each value and error bar represents the mean of quadruplicate samples and its standard deviation.

Supplementary References

(31) A. R. Spurr *Ultrastruc. Res.* 1969, **26**, 31.

(32) D. X. Oh, S. Shin, C. Lim, D. S. Hwang, *Materials* 2013, **6**, 3826.

(33) J. S. Temenoff, K. A. Athanasiou, R. G. Lebaron, A. G. Mikos, *J. biomed. Mater. Res.* **2002**, 59, 429.
